# Age-associated epigenetic drift: implications, and a case of epigenetic thrift?

**DOI:** 10.1093/hmg/ddt375

**Published:** 2013-08-04

**Authors:** Andrew E. Teschendorff, James West, Stephan Beck

**Affiliations:** 1 Statistical Cancer Genomics and; 2 Medical Genomics, UCL Cancer Institute, University College London, Paul O'Gorman Building, 72 Huntley Street, London WC1E 6BT, UK and; 3 Centre for Mathematics and Physics in the Life Sciences and Experimental Biology, University College London, London WC1E 6BT, UK

## Abstract

It is now well established that the genomic landscape of DNA methylation (DNAm) gets altered as a function of age, a process we here call ‘epigenetic drift’. The biological, functional, clinical and evolutionary significance of this epigenetic drift, however, remains unclear. We here provide a brief review of epigenetic drift, focusing on the potential implications for ageing, stem cell biology and disease risk prediction. It has been demonstrated that epigenetic drift affects most of the genome, suggesting a global deregulation of DNAm patterns with age. A component of this drift is tissue-specific, allowing remarkably accurate age-predictive models to be constructed. Another component is tissue-independent, targeting stem cell differentiation pathways and affecting stem cells, which may explain the observed decline of stem cell function with age. Age-associated increases in DNAm target developmental genes, overlapping those associated with environmental disease risk factors and with disease itself, notably cancer. In particular, cancers and precursor cancer lesions exhibit aggravated age DNAm signatures. Epigenetic drift is also influenced by genetic factors. Thus, drift emerges as a promising biomarker for premature or biological ageing, and could potentially be used in geriatrics for disease risk prediction. Finally, we propose, in the context of human evolution, that epigenetic drift may represent a case of epigenetic thrift, or bet-hedging. In summary, this review demonstrates the growing importance of the ‘ageing epigenome’, with potentially far-reaching implications for understanding the effect of age on stem cell function and differentiation, as well as for disease prevention.

## INTRODUCTION

DNA methylation (DNAm) is a key epigenetic mark of regulatory potential (1) affecting mostly (but not exclusively) cytosines in a CpG context (2). The observation that DNAm in normal cells is altered as a function of age has a relatively long history, with early studies already reporting age-associated changes affecting a small number of individual gene loci, notably high CpG density promoters of important cancer genes such as *IGF2* (3) and *ESR1* (4; see also 5,6). Global (genome-wide) hypomethylation with age was also noted early on (7), with subsequent confirmation in a longitudinal study of global DNAm patterns (8). It was also observed that monozygotic (MZ) twins exhibit epigenetic divergence in DNAm patterns that increases with age and differences in lifestyle (9). The advent of novel biotechnologies, allowing highly accurate assessment of DNAm levels across at least tens of thousands of CpG sites (10), have allowed more recent studies to test and confirm these earlier observations. For instance, using Illumina Infinium 27K arrays (11), in which probes map mainly to gene promoters, three separate studies (12–14) have demonstrated that age-associated increases in DNAm happen preferentially at the promoters of key developmental genes, notably those bivalently marked in embryonic stem cells (15) and which are often also marked by the Polycomb Repressive Complex (PRC2) and thus generally referred to as PolyComb Group Targets (PCGTs) (16). Many of the bivalent genes/PCGTs encode known tumour-suppressors and transcription factors (TFs) necessary for differentiation. These data were derived from measuring DNAm in normal tissue, specifically in human whole-blood tissue (13,14), as well as in murine intestinal cells (12). Two more recent studies (17,18), using the more comprehensive and unbiased Illumina 450K arrays (19), have further confirmed that age-associated hypermethylation happens preferentially at high CpG density promoters, which often reside upstream of key developmental genes such as PCGTs. These studies have also confirmed that the majority of changes in the genome involve loss of methylation affecting CpG sites located in low CpG density regions, in line with the fact that most of these sites start out as methylated (17). Thus, it would appear that the machinery responsible for maintaining normal DNAm patterns becomes gradually deregulated with age, leading to deviations from a normal epigenetic state, a process we call epigenetic drift (18). Next, we briefly discuss the potential biological, functional, clinical and evolutionary significance of this age-associated epigenetic drift.

## TISSUE-SPECIFIC AND TISSUE-INDEPENDENT AGE-ASSOCIATED DNAm SIGNATURES

One natural question that arises in the context of the observed epigenetic drift is whether this phenomenon is tissue-specific. Back in 2007, Issa and co-workers (20) already noted that age-related DNAm changes affecting certain genes were only seen in specific tissues. In trying to fully address this question, it is important to appreciate the complex nature of the profiled tissues, which often encompass a variety of different cell types. Thus, if the cellular composition of tissues alters with age, then this could lead to age-associated DNAm signatures which purely reflect these underlying changes in cell type. Indeed, a number of studies have now shown that some of the age-associated DNAm changes seen in whole-blood tissue, especially those involving hypomethylation, can be related to an age-associated skew in blood cell-type proportions, specifically in the relative proportion of myeloid to lymphoid cells (21–23). This is consistent with reports of global hypomethylation as being associated with commitment to the myeloid lineage (24). Thus, age-associated DNAm signatures reflecting changes in tissue composition are likely to be tissue-specific, and indeed, these generally do not validate in other tissue types (25). Therefore, changing cellular composition is a major confounder when assessing DNAm changes, and, in response to this, statistical methods aimed at dissecting this cellular heterogeneity have recently emerged (22,26). Application of these methods will be key since effect sizes associated with age and with other EWAS phenotypes could be small (27). Indeed, these methods have already been shown to have a dramatic impact on statistical inference and significance estimates (28).

**Figure 1.**
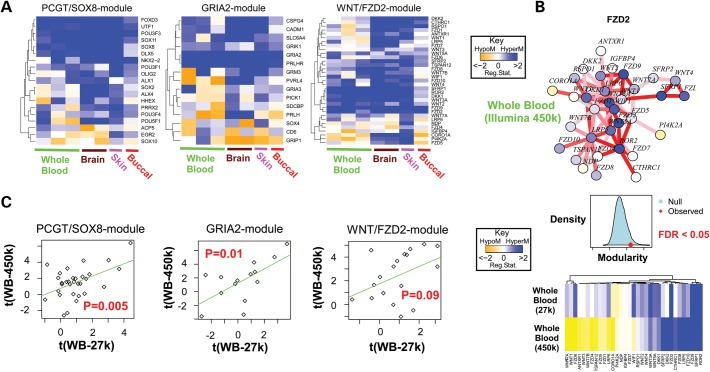
Tissue-independent age-associated DNAm signatures. (**A**) Three gene modules derived using Illumina 27K arrays from whole blood with promoters undergoing significant changes in DNAm with age, demonstrating consistency across different tissue types (independent whole-blood data sets, brain, skin and buccal cells) (25). (**B**) FZD2/WNT-signalling interactome module from West *et al*. (25) with age-associated directional DNAm changes as derived from the Heyn *et al*.'s (17) whole-blood data (Illumina 450K arrays). Middle panel shows the validation of the modularity, i.e. the interactome hotspot nature of the module (25) in the 450K data set, and lower panel shows the overall consistency between the 27 and 450K sets, especially for those gene promoters undergoing significant hypermethylation with age. (**C**) Validation of the directional age-associated DNAm changes (*t*-statistics) between the whole-blood (WB) training set (27K) and the independent whole-blood 450K data set of Hannum *et al*. (18).

Interestingly, however, a number of recent studies have also demonstrated age-associated DNAm signatures that are largely independent of tissue type (12–14,29). For instance, a DNA hypermethylation signature consisting of 69 CpGs mapping to promoters of PCGTs was validated not only in whole blood but also in normal tissue from the cervix, lung and even in ovarian cancer cells (13). A separate study derived a similar signature, enriched for bivalently marked genes, which was then validated in purified CD4+ T-cells and CD14+ monocytes, thus effectively discarding changing blood cell-type composition as the underlying reason for these signatures (14). A recent meta-analysis focusing on brain and blood tissue also concluded that a large proportion of age-associated DNAm changes are common to both tissue types, with important implications for studying epigenetic effects in diseases like Alzheimer's disease (29). Another more recent study used a systems approach to identify interactome hotspots of age-associated differential methylation, which were found to target stem cell differentiation pathways and to be independent of tissue type (25) (Fig. 1A). These age-associated interactome modules, derived with Illumina 27K arrays, have also been validated in data generated using Illumina 450K arrays (Fig. 1B and C). In summary, although it is hard to absolutely discard age-associated changes in tissue composition as the underlying mechanism of these common tissue-independent signatures, following Occam's Razor, the evidence does point towards the simplest explanation, which is that gradual age-associated accumulation of DNAm changes does occur independently of cell type.

## DNAm-BASED AGE PREDICTORS

The observation that a number of age-associated DNAm signatures validate consistently across so many different tissue types is remarkable, given that analogous robust molecular signatures at the copy-number, mutational or transcriptomic levels have not been reported, or at least not at the same level of consistency as seen for DNAm. In fact, a meta-analysis of age-associated gene expression changes only reported marginal statistically significant agreement across studies, although, interestingly implicating genes with roles in metabolism and DNA repair (30). Telomere attrition and other molecular features such as T-cell DNA rearrangements can predict age, but the reported prediction accuracies are not high (31–34). More recently, a study reported mosaic copy-number changes with age, yet whether specific age-predictive copy-number-based signatures can be derived is unclear (35). In contrast, at least three separate studies have now reported DNAm -based age predictors (18,36,37). For instance, in Hannum *et al.* (18), a DNAm-based age signature derived in whole blood could predict the age of independent blood samples with a median absolute deviation of only ±5 years. The authors further noted that this signature was highly correlated with age in other tissue types, but that highly accurate absolute age estimates could be achieved only if the parameters were retrained (18). It, therefore, seems likely that the high predictive accuracy attained by tissue-specific signatures is driven partly, if not entirely, by tissue-specific effects. Mechanistically, if changes in cell-type composition with age are highly certain and tissue-specific, then this would lead to corresponding highly accurate tissue-specific age predictors. This seems particularly relevant in the haematopoietic system where an age-associated skew towards the myeloid lineage has been observed (38–40). Thus, it remains to be seen whether tissue-independent age-associated DNA methylation signatures can achieve the predictive accuracy of tissue-specific DNAm signatures as reported in, e.g., Hannum *et al.* (18). Irrespective of the underlying biological mechanism, the high accuracy of some of the DNAm-based age predictors derived so far already promises some exciting novel applications, for instance in forensic science it has been suggested that they could be used to determine the approximate age of a suspect or victim from DNA samples collected at the crime scene (18,36). However, before this or other applications may be considered, it will be important to validate existing and novel DNAm-based age predictors more extensively, using truly independent cohorts. In doing so, particular care must also be taken in relation to technical confounding factors (e.g. batch effects), as these could easily skew or bias results (41–43).

Most of the age-predictive models reported so far are linear univariate or multivariate models, which assume that the rate at which age-associated DNAm changes accumulate is constant. This ageing rate has been shown to depend on genetic factors, and notably also on sex, with men exhibiting a faster rate (18). It remains to be seen, however, whether the rate of change is indeed constant, or whether instead a non-linear model could more accurately reflect how changes accumulate with age. For instance, a recent DNAm study performed on whole-blood samples from a paediatric population, consisting of boys aged between 3 and 17 years, showed that even at this young age, DNAm changes associated with age can be detected and, intriguingly, that these early changes account for most of the variation seen in the adult population (44). Thus, the authors of this study suggested a log-linear model to describe age-associated DNAm changes. However, not being a longitudinal study, and with the samples from the adult populations coming from different cohorts and generated by independent groups, this analysis could be subject to the usual caveats of confounding factors (41,43). A recent longitudinal twin study comparing buccal DNAm profiles of newborn twins with those at 18 months of age noted significant (3% differences at 12 months) age-associated DNAm changes (45). This is noteworthy in light of cross-sectional studies reporting typically 10% or at most 25% changes in DNAm across wider age ranges encompassing several decades (13,18). Thus, put together, the data from Alisch *et al*. (44) and Martino *et al*. (45) seem to suggest that age-associated epigenetic drift kicks in immediately after birth and may be particularly prominent during pre-puberty. These observations are surprising, yet also very interesting in light of studies proposing that the epigenome might be particularly sensitive to environmental stressors (e.g. nutrient deprivation) during pre-puberty (46,47). Thus, cumulative age-associated exposure to environmental factors during early life could be an important driver of epigenetic drift.

## IMPLICATIONS OF AGE-ASSOCIATED DNAm FOR AGEING, STEM CELL BIOLOGY AND REPROGRAMMING

It is a striking observation that it is precisely the tissue-independent age DNAm signatures that also seem to validate in stem cell populations (13,25). For instance, an age-associated DNAm signature, derived from whole-blood tissue and enriched for PCGTs, was validated in bone marrow-derived mesenchymal stem cells (MSCs) from eight donors spanning a wide age range (13). A similar signature was also observed to be present in haematopoietic progenitor cells (HPCs) (48). Other tissue-independent age signatures have also been validated in MSCs and in HPCs (25). Thus, it would appear that the generic epigenetic drift observed across all tissue types may be driven by changes in underlying long-lived stem cells, thus explaining why these age-associated changes can be seen in differentiated cell populations with a high turnover rate such as the haematopoietic system.

If epigenetic drift does indeed occur in stem cells, then this drift could, over time, affect stem cell function. Supporting this possibility, a recent bioinformatics study (25) identified tissue-independent age-associated differential methylation interactome hotspots, specifically targeting a number of stem cell differentiation pathways (Fig. 1). One of these hotspots was enriched for stem cell TFs, including *UTF1*, *SOX8* and *SOX2*, with their promoter CpGs gradually becoming hypermethylated with age. *UTF1* is a TF necessary for the differentiation of human embryonic stem cells and has also recently been implicated as an important marker of reprogramming efficiency (49). Another age-associated network hotspot was found to be enriched in genes involved in WNT signalling, a pathway of key importance for normal differentiation of stem cells (50). Interestingly, age-associated changes in WNT-signalling activity are well documented (51,52), yet whether this is due to epigenetic deregulation is unclear. Interpretation is complicated by the fact that the promoters of both negative regulators and receptors of this pathway all become hypermethylated with age, hence the net effect of these changes is hard to predict.

**Figure 2.**
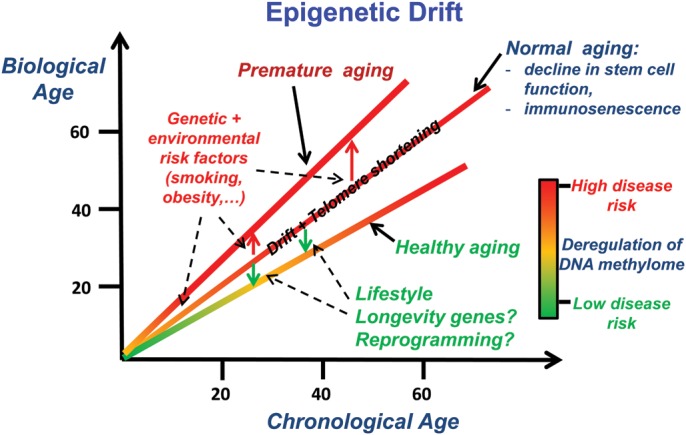
Putative effects of epigenetic drift. In normal ageing, whereby an individual is not significantly exposed to disease risk factors and does not have an unfavourable genotype, the deregulation of DNAm happens only gradually and possibly in a linear fashion, as demonstrated by highly accurate age-predictive linear models (18). In contrast, an individual exposed to risk factors, either environmental or genetic, may experience an aggravated or premature ageing profile, characterized by an abnormally higher deregulation of DNAm patterns, increasing the risk of age-related diseases like cancer or diabetes. One can further hypothesize that individuals with a favourable genotype (longevity genes) and with a healthy lifestyle may preserve a more intact epigenome and hence experience longevity. Reprogramming of aged cells into iPSCs and regeneration of differentiated cells may provide a mechanism for epigenetic rejuvenation. In addition to epigenetic drift, telomere shortening has been associated with ageing, age-associated stem cell dysfunction and disease risk factors.

Importantly, a number of experimental studies have shown that the age-associated DNAm changes seen in stem cells may underlie the observed decline in stem cell function, for instance in the case of MSCs (53) and myogenic stem cells (54). Further important support for this hypothesis was recently provided by a study showing that DNAm changes associated with haematopoietic stem cell (HSC) ontogeny happen preferentially at PRC2 targets, and in particular at genes that would normally be expressed in differentiated progeny (55). In fact, this study also reported silencing (through DNA hypermethylation) of key TFs needed for cell-lineage specification in aged HSCs, although the overall genome-wide correlation between DNAm and gene expression was very low, suggesting that most DNAm changes at PRC2 targets in HSCs only affect their transcriptional competency when passed on to downstream progeny (55). In summary, age-associated DNAm changes in adult stem cells possibly underpins the observed decline in stem cell function (Fig. 2), including the observed myeloid skewing of the ageing haematopoietic system (55) and immunosenescence (56).

Reprogramming of adult differentiated cells into induced pluripotent stem cells (iPSC) is accompanied by widespread changes in the DNAm landscape (57). Interestingly, loci undergoing age-associated DNAm changes have been observed to overlap significantly with those undergoing changes in reprogramming experiments (57,58). Thus, an intriguing and exciting possibility, suggested now by a number of studies and reviewed in Rando *et al.* (59), is that of ‘epigenetic rejuvenation’, whereby age-driven accumulation of DNAm changes could be rewound and reset to zero, thus resembling the DNAm patterns of embryonic stem cells.

## AGE-ASSOCIATED DNAm, DISEASE RISK AND CAUSALITY

Age-associated DNAm targeting the promoters of key tumour-suppressor genes in normal tissue has long been noted (5,7). Moreover, age-associated epigenetic divergence has been observed in MZ twins, with the drift proportional to the environmental divergence as measured by differences in lifestyle and time spent apart (9), suggesting that epigenetic drift could underlie their observed disease discordancy (60,61). Using Illumina beadarrays, a recent study demonstrated a highly statistically significant overlap between genes undergoing age-associated hypermethylation in their promoters and gene promoters undergoing hypermethylation in cancer (13), both involving preferentially PRC2 targets (62–64). Furthermore, one also observes strong overlaps of these genes with gene promoters characterized by DNAm changes associated with specific cancer risk factors [e.g. smoking (65), inflammation (66–68), obesity (69) and viral oncoprotein expression (70)]. Thus, age, cancer and cancer risk factor DNA hypermethylation signatures all seem commonly enriched for bivalent marks and PCGTs. However, not all DNAm changes associated with known cancer risk factors correlate with those seen in ageing—for instance, this seems to be the case for sunlight/UV exposure (71). In contrast to DNAm, it is only more recently that the age-associated mutational burden in normal tissue has been assessed (72), and therefore it is yet unclear if age- and cancer-associated mutational signatures overlap to the same degree as observed at the DNAm level.

Given that (i) age-associated DNAm changes are seen in normal tissue, (ii) that these are shared with those associated with cancer risk factors and (iii) that age is the strongest demographic risk factor for cancer (73), it is thus entirely plausible that these DNAm changes could predispose to cancer and thus be used for early detection or risk prediction. Supporting this, an age-associated DNAm signature enriched for PCGTs was found to be aggravated in intraepithelial neoplasias of the cervix, a pre-invasive cancer lesion (13). In a subsequent study that used a novel statistical risk-prediction algorithm based on epigenetic variable outliers, DNAm profiles measured in cytologically normal cervical swabs collected 3 years in advance of morphological transformation were shown to predict the future risk of a high-grade cervical intraepithelial neoplasia (CIN2+) with a low, yet statistically significant, AUC of approximately 0.64 (74). Importantly, the CpG sites making up this risk classifier were shown to be associated with age in normal cervix and other normal tissue types including blood, as well as being highly differentially variable between individuals at different risk of developing CIN2+ (74). Supporting this, inter-individual variable DNAm sites have also been shown to correlate with disease predisposition (75) and to be enriched for bivalent/PCGT genes (76).

The overlap between DNAm changes associated with age in normal tissue with those conferring risk of cervical cancer is intriguing. Interestingly, overexpression of an HPV-associated viral oncoprotein has recently been shown to lead to widespread DNA hypermethylation at promoters of PRC2 targets (70). This suggests that at least some of the DNAm changes associated with the risk of cervical cancer are likely to have been caused by HPV infection. In this regard, it is important to recall, however, that HPV infection, although necessary, is not a sufficient factor for cervical cancer. Hence, it is possible that age-associated epigenetic drift, possibly linked with a cumulative exposure to other risk factors, contributes to disease predisposition and that further HPV-induced epigenetic alterations then synergize with these to allow initiation of morphological transformation (74).

The intriguing link between age-associated epigenetic drift and the changes seen in cancer and in precursor cancer lesions suggests a causal contributing role for DNAm in disease initiation and may also extend to other diseases. Indeed, a recent study analysing DNAm profiles of patients with Hutchinson–Gilford Progeria and Werner Syndrome, a premature ageing condition, concluded that DNAm changes may play a key causal or mediating role in these diseases (77). In fact, in those patients where the syndrome could be linked to genetic mutations in known causal genes (*LMNA* and *WRN*), aberrant DNAm profiles showed a remarkable overlap with those associated with age. Interestingly, DNAm changes, although distinct ones, were also observed in those patients not carrying the causal genetic mutation, suggesting that DNAm changes could play a causal role in these subsets of patients. Further support for a causal role of DNAm in mediating disease risk was provided by a recent EWAS study, which identified a number of methylation quantitative trait loci associated with rheumatoid arthritis (28). Finally, a number of epidemiological studies have also linked epigenetic changes with overall stress levels, itself a major risk factor for neurological diseases (78).

## AGE-ASSOCIATED EPIGENETIC DRIFT: FUTURE DIRECTIONS

It is clear that the epigenome is altered by an age-associated epigenetic drift, whereby normal methylation patterns become deregulated with age. High CpG density promoters, and in particular those mapping to developmental genes, acquire methylation, whereas CpGs located outside these regions tend to lose methylation with age. However, a number of key questions require urgent attention. First, what is the precise biological mechanism (or mechanisms) leading to the deregulation of the normal DNAm patterns? While age-dependent expression of DNA methyltransferase genes has been reported (79), other epigenetic modulators (e.g. Sirtuins) may likely play an equally or even more important role (80–82). Methyl-binding domain proteins (e.g. *MBD4*) also seem implicated in modulating the rate of epigenetic drift (18). More fundamentally, it has been proposed that long-term deregulation of DNAm patterns occurs in response to spontaneous loss of histone modifications that happen on shorter timescales and in direct proportion to the number of cell divisions (55,83). To elucidate the mechanisms of regulation, it might help to investigate the degree of spatial stochasticity of age-associated DNAm changes. Besides CpG density, fairly little is known as to which other DNA sequence features may effect these age-related changes. Thus, it would be interesting to see whether age-associated methylation changes ‘cluster’ spatially as is observed, for instance, in cancer (84,85), or if instead they implicate a higher proportion of ‘singleton CpGs’, i.e. those that exhibit solitary DNAm changes and which are, therefore, less likely to be of functional significance. Heyn *et al.* (17) reported an overall loss of spatial correlations in the DNA methylome of centenarians, yet another study did report finding extended age-associated DMRs (aDMRs) (45). How frequent aDMRs are and how they compare with cancer DMRs in terms of their spatial correlative patterns remain to be seen. The precise pattern of CG dinucleotides in sequences affected by age-associated DNAm may also point to which epigenetic enzymes might be implicated (86). Alternatively, does one observe preferential enrichment of specific TF motifs among the sites that acquire age-associated DNAm changes, which would then point to the importance of specific TFs in mediating this age-associated deregulation of DNAm patterns, analogous to the TF-mediated redistribution of DNAm patterns one observes in response to stem cell differentiation and disease (87,88).

A second pressing question relates to the functional consequences of epigenetic drift, since it would appear that the association between age-driven DNAm and gene expression changes is, at the very best, only marginal (18,25,55). Moreover, it could well be that the weak association between DNAm and gene expression observed in whole blood (18) is entirely driven by underlying changes in blood cell-type composition with no direct effect on cell function. Thus, it will be interesting to perform comprehensive paired DNAm and transcriptomic profiling of specific genes/pathways (e.g. WNT-signalling pathway) undergoing age-associated DNAm changes in a large number of cell-purified samples to assess the functional impact of the epigenetic changes. It is very likely that only a very small fraction of the age-associated epigenetic drift is of functional consequence, with the few functional changes ultimately affecting key transcriptional regulators, thus compromising stem cell differentiation (55) or predisposing cells to neoplastic transformation (74).

A third key outstanding question is the dissection of age-associated DNAm changes that grow with ‘chronological age’, reflecting the number of cell divisions of long-lived stem cell populations, from the age-associated DNAm changes that may result from cumulative exposure to environmental risk factors, as well as from the changes that may accumulate with age in response to underlying genetic risk factors (8,18) (Fig. 2). Two longitudinal studies, one on MZ twins (45) and another involving families from an Icelandic cohort (8), have shown the importance of genotype in influencing the DNAm changes seen with age. Considerations of these separate components thus lead to the notion of a ‘biological’ age, as measured by the overall deregulation of DNAm in the genome of an individual, and which may be indicative of an overall prospective disease risk (Fig. 2). Epigenetic studies in model organisms where (isogenic) animals can be kept under controlled environmental conditions, allowing, for instance, a sustained and constant exposure to risk factors, seem key in order to help dissect the relative contributions of the intrinsic and extrinsic ‘epigenetic clocks’ in determining the biological age of the organism. Although Beerman *et al.* (55) studied DNAm changes during HSC ontogeny and concluded that most of the age-associated hypermethylation at PRC2 targets was determined by the proliferative history of the HSCs (i.e. the intrinsic clock), these were not cells that had been exposed to the effects of environmental risk factors, as it might happen, for instance, under inflammatory conditions.

A fourth key question concerns the relative importance of epigenetic drift in comparison with other age-associated biological effects, most notably the well-known shortening of telomeres with age (31,89–91). Curiously, age-associated epigenetic drift and telomere shortening share many similar properties: both processes are influenced by genotype (18,92,93), both have been proposed to lead to stem cell dysfunction (55,94), both are aggravated in men compared with women (18,91,92), both are tissue-independent phenomena (13,95), and both have been linked to disease, disease risk and disease risk factors (13,18,74,96–100). For instance, a recent study reported seven genetic variants associated with leucocyte telomere length (LTL), with inter-individual variation in LTL being associated with cancer and other age-related diseases (92). Thus, both age-associated epigenetic drift and telomere shortening have been proposed as markers of biological ageing (13,18,92,101). In this regard, it is important to note that although epigenetic drift seems to outperform telomere length as a predictor of chronological age, it is the estimated deviations between predicted (i.e. biological) and chronological age that are potentially of most interest and which may account for the observed variation in disease risk. Thus, it remains to be seen whether epigenetic drift or telomere attrition is a more relevant marker of biological ageing. Matched LTL and DNAm data for MZ twin pairs discordant for disease status or for exposure to environmental risk factors could elucidate the relative contributions of these two biological processes to the biological ageing and disease risk phenotype.

## EPIGENETIC DRIFT: A CASE OF EPIGENETIC THRIFT?

Finally, it is of interest to discuss the potential evolutionary significance of age-associated epigenetic drift. One attractive framework in which to interpret epigenetic drift is in the context of evolutionary theories of ageing. One competing theory argues that ageing emerged early in evolution as a means of controlling population size (102,103). It is conceivable that during times of limited resources or famine, which would have been frequent in early living history, overpopulation could lead to resource depletion and severe risk of mass extinction. Thus, in our ancestral species, natural ‘group’ selection could have favoured genetic and epigenetic mechanisms that promote ageing, with the damaging effects of these mechanisms only kicking in after the reproductive period, thus allowing new improved gene pools to take over (104) and keeping overall populations at a stable and sustainable level (102). This viewpoint is supported by a related idea, grounded on evolutionary mathematical principles, and referred to as highly optimized tolerance (105). This evolutionary theory proposes that biological organisms, and multi-cellular species in particular, represent states of highly optimized tolerance, providing robustness to common perturbations, but simultaneously, and also inevitably, implicating costly trade-offs, such as an increase in fragility, as exemplified by the ageing phenotype. Thus, it is tempting to speculate that epigenetic drift is one possible mechanism contributing to the ageing phenotype (e.g. through a decline in stem cell function) and to an associated increased risk of disease and death (e.g. through increased predisposition to cancer or other age-related diseases, and possibly mediated by immunosenescence). As mentioned earlier, another mechanism could be telomere shortening (96), and so both telomere shortening and epigenetic drift may be seen as providing an evolutionary benefit to the species as a whole, by managing population dynamics through ageing and increased fragility.

There are a number of other important observations which further support a role for epigenetic drift in human evolution. For instance, a recent study has shown that epigenetic drift does not happen randomly in the context of the human interactome, but that it preferentially affects genes of low connectivity and centrality (106). Thus, genes carrying out integral housekeeping and cellular functions, and which are generally of high connectivity and centrality, appear to be more protected from epigenetic drift. Since epigenetic drift kicks in straight after birth (45) and is prominent even in paediatric populations (44) (i.e. well before the reproductive period), it is tempting to speculate that drift affecting highly integral and essential genes would be weeded out by natural selection. In contrast, natural selection would not be able to efficiently weed out the epigenetic drift targeting the non-essential and less integral genes, since the main effects of drift at these genes only show up after the reproductive age. Indeed, as argued earlier, epigenetic drift targeting non-essential genes may even be selected for as a mechanism underlying ageing and increased fragility, which are necessary for population control.

The observation that epigenetic drift is prominent in early life (44,45), and that associated epigenetic changes could be heritable (107–110), further suggests that epigenetic drift may represent another example of ‘thrift’, a term first coined by James Neel in the genetic context (111), whereby genes that would have conveyed an evolutionary advantage to our ancestral species would now lead to an opposing, apparently detrimental, effect in today's resource-rich society. Indeed, the genetic thrift hypothesis has recently been invoked to explain the current obesity and metabolic disease epidemics (112), according to which, genes favouring our ancestors, for instance, in promoting fat storage in anticipation of possible famines, would now have detrimental effects. Interestingly, epigenetic (or phenotype) thrift has also been proposed as the underlying mechanism to explain the increased incidence of diabetes and cardiovascular disease among people born during the 1944 Dutch Winter Famine (113,114). A compromised resource-depleted *in utero* environment could lead to epigenetic deregulation of metabolic genes to promote a more favourable metabolic state, which in a food-rich environment, however, would only be detrimental (114). Thus, if epigenetic changes are heritable, this would allow epigenetic drift to quickly shape phenotypes and evolution.

In summary, it is tempting to speculate that DNAm changes associated with epigenetic drift, which may be heritable, represent a case of epigenetic thrift, or perhaps even a case of evolutionary bet-hedging (83,105,115), contributing to both phenotypic diversity and the ageing phenotype, in a way that optimized evolutionary adaptation and species survival in the face of potential uncertain adversities.

*Conflict of Interest statement*. None declared.

## FUNDING

A.E.T. is supported by a Heller Research Fellowship. J.W. is supported by an EPSRC/BBSRC PhD studentship awarded to CoMPLEX. S.B. was supported by the Wellcome Trust (WT084071) and a Royal Society Wolfson Research Merit Award (WM100023). Funding to pay the Open Access publication charges for this article was provided by the Wellcome Trust (WT084071).
